# *Streptomyces malaysiense* sp. nov.: A novel Malaysian mangrove soil actinobacterium with antioxidative activity and cytotoxic potential against human cancer cell lines

**DOI:** 10.1038/srep24247

**Published:** 2016-04-13

**Authors:** Hooi-Leng Ser, Uma Devi Palanisamy, Wai-Fong Yin, Kok-Gan Chan, Bey-Hing Goh, Learn-Han Lee

**Affiliations:** 1 School of Pharmacy, Monash University Malaysia, 47500 Bandar Sunway, Malaysia; 2 Biomedical Research Laboratory, Jeffrey Cheah School of Medicine and Health Sciences, Monash University Malaysia, 47500 Bandar Sunway, Selangor Darul Ehsan, Malaysia; 3 Division of Genetics and Molecular Biology, Institute of Biological Sciences, Faculty of Science, University of Malaya, 50603 Kuala Lumpur, Malaysia; 4 Center of Health Outcomes Research and Therapeutic Safety (Cohorts), School of Pharmaceutical Sciences, University of Phayao, Phayao, Thailand

## Abstract

Actinobacteria from the unique intertidal ecosystem of the mangroves are known to produce novel, bioactive secondary metabolites. A novel strain known as MUSC 136^T^ (=DSM 100712^T^ = MCCC 1K01246^T^) which was isolated from Malaysian mangrove forest soil has proven to be no exception. Assessed by a polyphasic approach, its taxonomy showed a range of phylogenetic and chemotaxonomic properties consistent with the genus of *Streptomyces*. Phylogenetically, highest similarity was to *Streptomyces misionensis* NBRC 13063^T^ (99.6%) along with two other strains (>98.9% sequence similarities). The DNA–DNA relatedness between MUSC 136^T^ and these type strains ranged from 22.7 ± 0.5% to 46.5 ± 0.2%. Overall, polyphasic approach studies indicated this strain represents a novel species, for which the name *Streptomyces malaysiense* sp. nov. is proposed. The potential bioactivities of this strain were explored by means of antioxidant and cytotoxic assays. Intriguingly, MUSC 136^T^ exhibited strong antioxidative activities as evaluated by a panel of antioxidant assays. It was also found to possess high cytotoxic effect against HCT-116 cells, which probably mediated through altering p53 protein and intracellular glutathione levels. Chemical analysis of the extract using GC-MS further affirms that the strain produces chemopreventive related metabolites.

There is an enormous need for novel chemotherapeutic agents in the ongoing battle against cancer, the global burden of which is steadily increasing^1^,^2^. Natural compounds are fast gaining interest as a potential source of new cancer treatments; it may serve as an alternative to overcome some of the current problems faced including debilitating side effects caused by drugs lacking in specificity^3^,^4^,^5^,^6^. Microorganisms are a prolific source of structurally diverse bioactive metabolites; some of which have been approved to be used as cancer chemotherapeutic agents^7^,^8^. Among microbes, the genus *Streptomyces* has garnered much interest from the scientific community due to its unrivaled capacity for the production of bioactive metabolites^7^. The genus *Streptomyces* was initially proposed by Waksman and Henrici^9^ in 1943; around the same time as the discovery of actinomycin from *Actinomyces antibioticus* (now *Streptomyces antibioticus*)^8^. Since the recognition of actinomycin D as an anticancer agent, many anticancer compounds have been isolated from *Streptomyces* species, including anthracyclines, bleomycin and mitosanes^7^,^8^.

In recent years, there has been increasing research investigating the biosynthetic potential of *Streptomyces* from underexplored areas, including mangrove forests^10^,^11^,^12^. The mangrove-derived microbial resources could potentially be an important source of compounds with pharmaceutical value, given that the changes in salinity and tidal gradient in the mangrove ecosystem may have resulted in the evolution of metabolic pathway producing valuable metabolites^11^,^12^,^13^. Numerous studies have discovered novel *Streptomyces* from mangrove environments, as demonstrated by the recent isolation of *Streptomyces sanyensis*^14^, *Streptomyces pluripotens*^15^, *Streptomyces gilvigriseus*^16^ and *Streptomyces mangrovisoli*^17^.

In this study, soil samples from the Tanjung Lumpur mangrove forest located in the east coast of Peninsular Malaysia was screened to identify novel strains exhibiting cytotoxic and antioxidant activities. Polyphasic approach revealed that MUSC 136^T^ strain represents a novel species of *Streptomyces* genus, for which the name *Streptomyces malaysiense* sp. nov. is proposed. The extract from this novel strain was demonstrated to have cytotoxic and antioxidant activities which was further characterized using gas chromatography-mass spectrometry (GC-MS). The findings of this research are anticipated to provide a strong basis for further in-depth molecular studies on the chemopreventive properties of the novel strain MUSC 136^T^.

## Results

### Phenotypic analyses of strain *Streptomyces malaysiense* MUSC 136^T^

**Figure 1:**
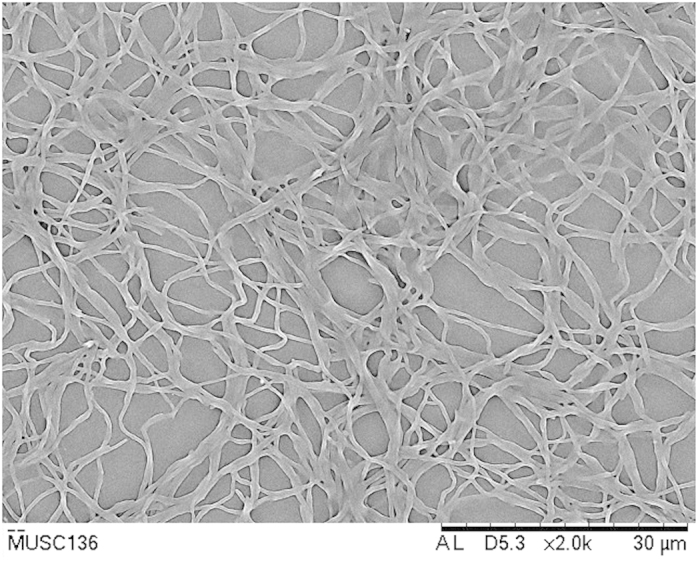
Scanning electron microscope of *Streptomyces malaysiense* MUSC 136^T^.

Strain MUSC 136^T^ grew well on ISP 2, ISP 5, ISP 6, ISP 7 agar, actinomycetes isolation agar, nutrient agar and starch casein agar after 7–14 days at 28 °C, moderately on *Streptomyces* agar and ISP 3 agar, and not at all on ISP 4 agar. The colors of the aerial and substrate mycelium were media-dependent (See Supplementary Table S1). The morphological observation of a 15-day-old culture grown on ISP 2 agar revealed abundant growth of both aerial and vegetative hyphae, which were well developed and not fragmented. These morphological features are consistent with assignment of the strain to the genus *Streptomyces*^18^ (Fig. 1, Table 1). Growth was found to occur at pH 6.0–7.0 (optimum pH 7.0), with 0–6% NaCl tolerance (optimum 0–2%) and at 26–40 °C (optimum 28–32 °C). Cells were found to be positive for catalase but negative for hemolytic activity and melanoid pigment production. Hydrolysis of soluble starch, tributyrin (lipase) and casein were found to be positive, but negative for hydrolysis of carboxymethylcellulose, chitin and xylan. Strain MUSC 136^T^ can be differentiated from closely related members of the genus *Streptomyces* using a range of phenotypic properties (Table 1). In chemical sensitivity assays, cells are resistant to aztreonam, fusidic acid, guanine HCl, lincomycin, lithium chloride, minocycline, nalidixic acid, niaproof 4, potassium tellurite, rifamycin RV, sodium bromate, sodium butyrate, 1% sodium lactate, tetrazolium blue, tetrazolium violet, troleandomycin and vancomycin.

### Phylogenetic and genomic analyses

**Figure 2:**
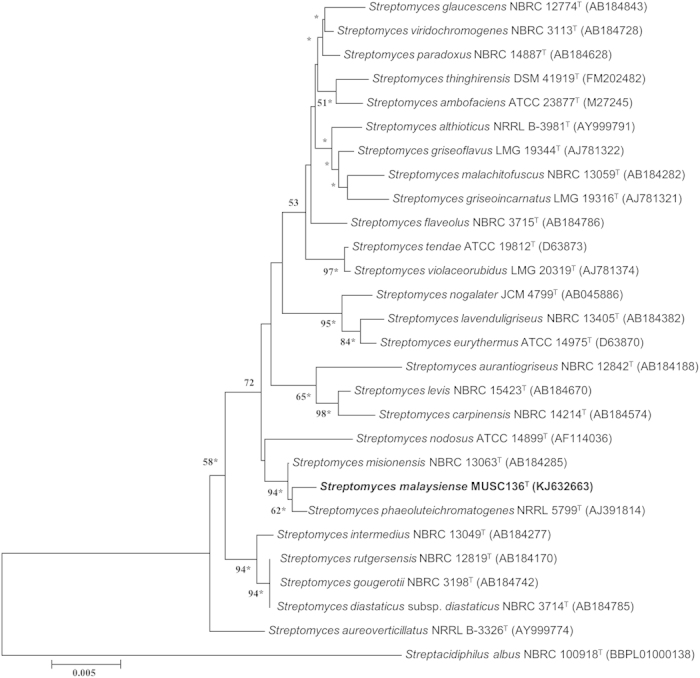
Neighbour-joining phylogenetic tree based on almost complete 16S rRNA sequences (1489 nucleotides) showing the relationship between strain MUSC 136^T^ and representatives of some other related taxa. Numbers at nodes indicate percentages of 1000 bootstrap re-samplings, only values above 50% are shown. Bar, 0.005 substitutions per site. Asterisks indicate that the corresponding nodes were also recovered using the maximum-likelihood tree-making algorithm. *Streptacidiphilus albus* NBRC 100918^T^ was used as an outgroup.

The nearly complete 16S rRNA gene sequence was obtained for strain MUSC 136^T^ (1489 bp; GenBank/EMBL/DDBJ accession number KJ632663) and phylogenetic trees were reconstructed to determine the phylogenetic position of this strain (Fig. 2 & See Supplementary Fig. S1). Phylogenetic analysis revealed that strain MUSC 136^T^ is closely related to *Streptomyces phaeoluteichromatogenes* NRRL 5799^T^ (99.6% sequence similarity) and *Streptomyces misionensis* NBRC 13063^T^ (99.6%), as they formed a distinct clade (Fig. 2). The 16S rRNA gene sequence analysis of strain MUSC 136^T^ showed the highest similarity to that of *Streptomyces misionensis* NBRC 13063^T^ (99.6%), followed by *Streptomyces phaeoluteichromatogenes* NRRL 5799^T^ (99.6%) and *Streptomyces rutgersensis* NBRC 12819^T^ (98.9%); sequences similarities of less than 98.9% were obtained with the type strains of other species of the genus *Streptomyces*. The DNA–DNA relatedness values between strain MUSC 136^T^ and *Streptomyces misionensis* NBRC 13063^T^ (46.5 ± 0.2%), *S. phaeoluteichromatogenes* DSM 41898^T^ (44.5 ± 0.4%) and *Streptomyces rutgersensis* NBRC 12819^T^ (22.7 ± 0.5%) were significantly below 70%, the threshold value for the delineation of bacterial species^19^.

The BOX-PCR results indicated that strain MUSC 136^T^ yielded a unique BOX-PCR fingerprint compared with the closely related type strains (See Supplementary Fig. S2). These results are in agreement with results of DNA-DNA hybridizations, which indicate that strain MUSC 136^T^ represents a novel species.

### Chemotaxonomic analyses

The fatty acid profile of strain MUSC 136^T^ and closely related type strains are shown in Table 2. The major cellular fatty acids in MUSC 136^T^ were identified as anteiso-C_15: 0_ (35.3%), iso-C_16: 0_ (12.4%), iso-C_15: 0_ (12.2%) and anteiso-C_17: 0_ (11.8%). The fatty acid profile of MUSC 136^T^ is consistent with those of closely related phylogenetic neighbours such as *S. misionensis* NBRC 13063^T^, *S. phaeoluteichromatogenes* DSM 41898^T^ and *S. rutgersensis* NBRC 12819^T^, which contain anteiso-C_15: 0_ (32.2–40.1%), iso-C_16: 0_ (10.3–17.7%), iso-C_15: 0_ (5.2–12.3%) and anteiso-C_17: 0_ (11.8–19.3%) as their major fatty acids (Table 2). Nevertheless, the fatty acid profile of MUSC 136^T^ was quantitatively different from those of these type strains; for example, although anteiso-C_15: 0_ (35.3%) was found to be predominant in strain MUSC 136^T^, the amount of anteiso-C_15: 0_ was much more (40.1%) in *S. misionensis* NBRC 13063^T^ (Table 2). The polar lipid analysis showed the presence of aminolipid, diphosphatidylglycerol, glycolipid, phospholipid, phosphatidylinositol, phosphatidylethanolamine, phosphoglycolipid and lipid. Differences in polar lipid profiles indicated that MUSC 136^T^ is different from related type strains; for instance, type strain *Streptomyces misionensis* NBRC 13063^T^ (See Supplementary Fig. S3b) was found to contain phosphatidylglycerol, lipids that were not detected in MUSC 136^T^ (See Supplementary Fig. S3a).

The cell wall of strain MUSC 136^T^ is of cell-wall type I as it contains LL-diaminopimelic^20^. The presence of LL-diaminopimelic has been observed in many other species of the genus *Streptomyces*^15^,^16^,^17^. The predominant menaquinones of strain MUSC 136^T^ were identified as MK-9(H_8_) (57%) and MK-9(H_6_) (20%). The detection of these predominant menaquinones is in agreement with report of Kim *et al.*^21^. The cell wall sugars detected were glucose and ribose. The G+C content of strain MUSC 136^T^ was determined to be 72.3 mol%; which is within the range of 67.0–78.0 mol% described for species of the genus *Streptomyces*^22^.

### Antioxidant activity of MUSC 136^T^ extract

The results show that MUSC 136^T^ extract demonstrated significant free radical scavenging activity (Table 3). At the highest tested concentration (2 mg/mL), the extract was able to scavenge 27.24 ± 1.91% of DPPH radicals and 27.87 ± 2.19% ABTS radicals. MUSC 136^T^ exhibited superoxide dismutase (SOD)-like activity as high as 68.27 ± 3.67% when tested at 2 mg/mL. Furthermore, the metal chelating activity (which ranged from 10.22–37.01% depending on concentration of extract) exhibited by MUSC 136^T^ further emphasizes its antioxidative potential by means of preventing transition metals from enhancing the generation of reactive oxygen species.

### Cytotoxic activity of MUSC 136^T^ extract

**Figure 3:**
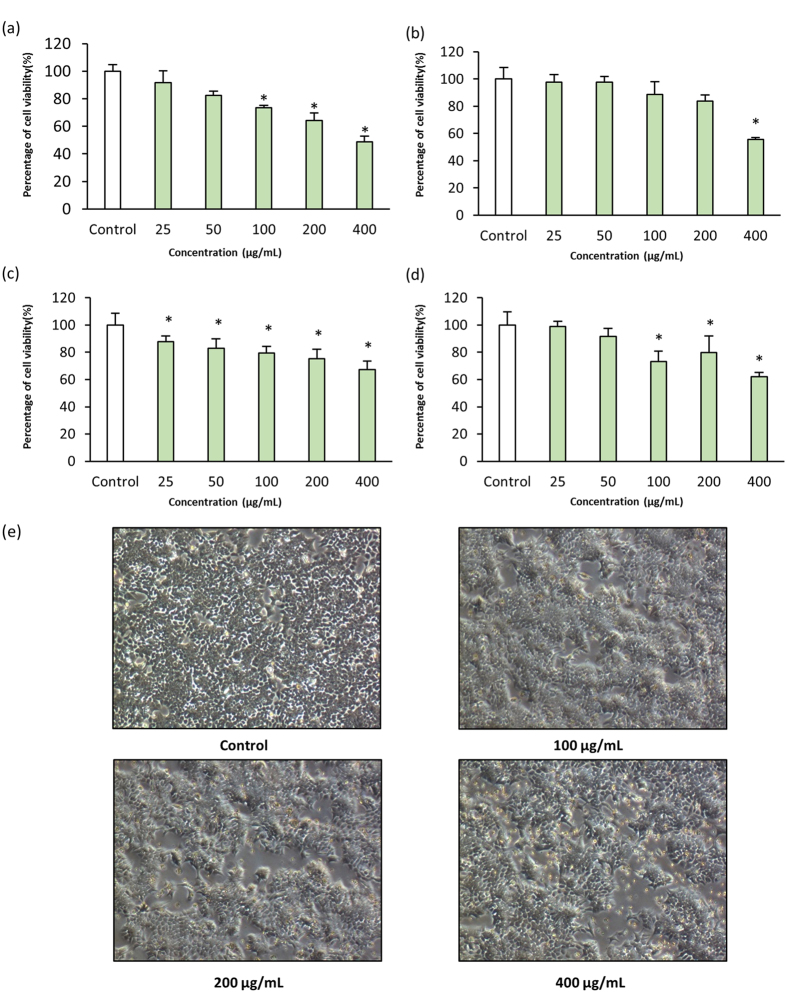
Cytotoxic activity of MUSC 136^T^ extract against human cancer cell lines. The measurement of cell viability was done using MTT assay. The graphs show cytotoxic effect of MUSC 136^T^ extract against (**a**) HCT-116, (**b**) Ca Ski, (**c**) A549, (**d**) HT-29. All data are expressed as mean ± standard deviation (n = 4) and analyzed using one-way analysis of variance (ANOVA). A difference was considered statistically significant when *p* ≤ 0.05. (**e**) Morphological studies show that after treatment with the extract, cells shrunk to smaller rounder cells and detached from the surface.

The cytotoxic potential of MUSC 136^T^ extract was tested against several human derived cancer cells (HCT-116, HT-29, Ca Ski and A549). The extract was found to be most toxic against HCT-116 with cell viability at 48.8 ± 4.1% when tested at 400 μg/mL (Fig. 3a). Additionally, a dose dependent effect was also observed when it was tested against HCT-116 cells. Second to HCT-116 cells, the cervical cancer cell line, Ca Ski showed decreased in cell viability to 55.6 ± 1.2% after treatment with MUSC 136^T^ extract (Fig. 3b). On the other hand, lung cancer cell line, A549 was found to be least sensitive to the extract treatment with cell viability at 67.1 ± 0.6% (Fig. 3c). Interestingly, differing levels of activity were seen among the colon cancer cells (HCT-116, Fig. 3a and HT-29, Fig. 3d) with a higher cytotoxic effect against HCT-116 cells.

### Morphological changes associated with apoptosis induced by MUSC 136^T^ extract

In order to visualize morphological changes in response to MUSC 136^T^ extract, HCT-116 cells were observed using phase contrast microscopy. Figure 3(e) shows features of control (untreated) cells which mostly consists of elongated attached cells. In contrast, cells treated with MUSC 136^T^ extract exhibited morphological alterations, including shrunken cell and detached from the surface.

### Measurement of intracellular glutathione (GSH) level and p53 protein expression following MUSC 136^T^ treatment on HCT-116 cells

**Figure 4:**
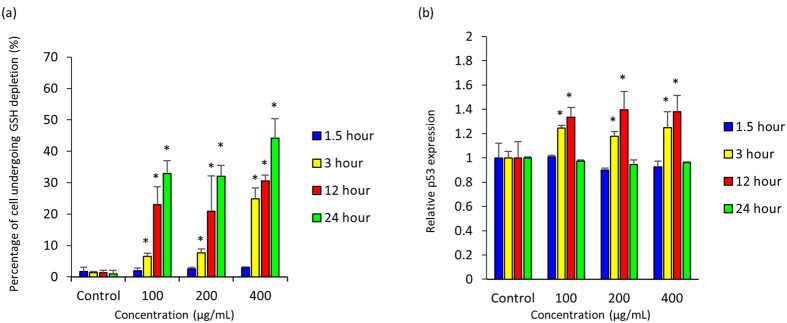
Effects of MUSC 136^T^ extract on (**a**) intracellular glutathione (GSH) content and (**b**) p53 protein in HCT-116 cells. All data are expressed as mean ± standard deviation (n = 3) and analyzed using one-way analysis of variance (ANOVA). A difference was considered statistically significant when *p* ≤ 0.05.

MUSC 136^T^ extract has caused a significant increase in number of cells experiencing in intracellular GSH depletion as seen in Fig. 4(a). The treatment of extract at the concentration of 400 μg/mL resulted in higher proportion of cells (44.11 ± 6.21%) undergoing GSH depletion as compared to control.

The p53 protein in HCT-116 cells was measured with flow cytometry at four time points after treated with MUSC 136^T^ extract (1.5, 3, 12, 24 hour); a significant increase was observed at 3 and 24 hour as compared to control (Fig. 4(b)). Overall, the p53 expression was found to be increasing 3 hour after MUSC 136^T^ extract treatment up to 12 hour as no significant changes was observed later when measured at 24 hour.

### GC-MC analysis of MUSC 136^T^ extract

GC-MS analysis revealed nine constituents of MUSC 136^T^ (Table 4) with the chemical structures (See Supplementary Fig. S4) Isomeric dihydro-methyl-furanone (**1**), 1-Pentadecene (**2**), Phenol, 2,5-bis (1,1-dimethylethyl)- (**3**), (3R,8aS)-3-methyl-1,2,3,4,6,7,8,8a-octahydropyrrolo[1,2-a]pyrazine-1,4-dione (**4**), 1,4-diaza-2,5-dioxobicyclo[4.3.0]nonane (**5**), Tetradecanoic acid, 12-methyl-, methyl ester (**6**), Pyrrolo[1,2-a]pyrazine-1,4-dione, hexahydro-3-(2-methylpropyl)- (**7**), Pentadecanoic acid, 14-methyl-, methyl ester (**8**) and Deferoxamine (**9**).

## Discussion

Cancer initiation and progression has been linked to oxidative stress where elevated amounts of free radicals are observed^22^. The accumulation or uncontrolled production of free radicals causes negative effects on critical cellular macromolecules such as membrane lipids, proteins and DNA. Free radical induced DNA mutations are known to increase cancer risk. Antioxidants play an important role in preventing the deleterious effects of free radicals in biological systems. In fact, they are also considered as one of the most promising cytotoxic agents against various human cancers. Continuous efforts have been directed at the search for more effective antioxidants from natural resources which could be developed into new therapeutic drugs^23^. *Streptomyces* species are prolific producers of bioactive metabolites with various biological activities, representing one of the most extensively studied microbial genus for novel drugs^9^,^15^,^17^. In the current study, the polyphasic approach revealed that strain MUSC 136^T^ merits assignment to a novel species in the genus *Streptomyces*. Thus, an attempt was made to explore the antioxidant potential of this novel strain utilizing DPPH, ABTS, metal chelating and superoxide anion scavenging assays. Our results revealed significant antioxidant potential of MUSC 136^T^ extract, which implies that the strain may produce bioactive secondary metabolites that could potentially reduce cancer risk and form the basis for further development as chemopreventive drugs.

Carcinogenesis is a multi-step process, involving activation of oncogenes and inactivation of tumor suppressor genes along with other complex interactions between host and tumor cells. p53 as a tumor suppressor protein has been studied extensively as a promising target for cancer treatment due to its involvement in cancer initiation and progression^24^,^25^. It is known to be involved in inducing growth arrest or apoptosis upon activation; dysfunction in the gene responsible for this tumor suppressor protein might result in unregulated cell division – a scenario which is commonly seen in cancer cells with mutated p53 genes such as colon cancer cell line HT-29. The current results revealed differing susceptibility of two colon cell lines, HCT-116 and HT-29 towards MUSC 136^T^ extract which could be due to the status of its tumor suppressor protein p53^26^,^27^. In view of this, the effect of MUSC 136^T^ extract on p53 tumor suppressor protein in HCT-116 was examined. The experiment demonstrated that the treatment of MUSC 136^T^ extract caused an increase in p53 protein expression in HCT-116 which may lead to the induction of apoptosis in this particular cell line. This prompted further analysis using morphological analysis. In brief, HCT-116 cells treated with MUSC 136^T^ extract exhibited morphological alterations associated with cell death including presence of shrunken cells and detached from the surface. Apart from morphological studies, intracellular glutathione (GSH) content was also evaluated as this ubiquitous non-protein thiol is essential for cell survival. Interestingly, the HCT-116 cells treated with the extract resulted in a decrease in intracellular GSH content. Since a decline in this molecule is known to trigger apoptotic signaling cascades particularly through activation of p53 signaling pathway, we postulate that it might potentially trigger the activation of p53-dependent apoptosis pathways^28^. However, further mechanistic studies on p53 associated cell death pathway would be needed to improve the understanding of the action target of MUSC 136^T^ extract.

Results from both antioxidant assays and *in vitro* cytotoxic screening suggest the presence of potent antioxidant(s) and anticancer agent(s) in the extract of MUSC 136^T^, thus chemical analysis using GC-MS was performed in order to provide clues as to their identities. This powerful analytical technique, which incorporates the separation power of gas chromatography and detection power of mass spectrometer has been widely used to identify the components of a mixture, including extracts of *Streptomyces* species^29^,^30^. In the current study, GC-MS detected nine compounds in the extract of MUSC 136^T^, with the majority of the compounds having been previously detected in marine-derived microorganisms including *Streptomyces* species^7^,^17^,^31^,^32^.

Pyrrolizidines, as natural occurring complex heterocyclic compounds, are known to exhibit a wide spectrum of bioactivity which includes antimicrobial, anticancer and antioxidant activity^9^,^31^. Two pyrrolizidines were identified in MUSC 136^T^ extract, 1,4-diaza-2,5-dioxobicyclo[4.3.0]nonane (**5**) and pyrrolo[1,2-a]pyrazine-1,4-dione, hexahydro-3-(2-methylpropyl)- (**7**). Interestingly, compound (**7**), which was recently identified in *Streptomyces cavouresis* KU-V39 was associated with cytotoxic activity of human cervical cancer^32^.The compound pyrrolo[1,2-a]pyrazine-1,4-dione, hexahydro-3-(2-methylpropyl)- (**7**), either alone or in combination with other compounds may be responsible for the cytotoxic activity observed in MUSC 136^T^ extract.

On the other hand, 1,4-diaza-2,5-dioxobicyclo[4.3.0]nonane (**5**) as a naturally occurring marine microbe-derived compound has been studied extensively for its antioxidant activity^17^,^33^. It was proven to be highly capable of scavenging or reducing the amount of free radicals when assessed with reducing power assay. It is postulated that the scavenging activity observed in MUSC 136^T^ extract could be due to the presence of 1,4-diaza-2,5-dioxobicyclo[4.3.0]nonane (**5**). Based on the assumption that antioxidants may influence survival of cancer cells^34^, the same compound 1,4-diaza-2,5-dioxobicyclo[4.3.0]nonane may contribute to the observed cytotoxic effect exhibited by MUSC 136^T^ extract.

The natural trihydroxamate, deferoxamine (**9**) which has been reported in several members of the *Streptomyces* family^35^,^36^ was also detected in the extract of MUSC 136^T^. As a drug listed on the World Health Organization’s List of Essential Medicines^37^, deferoxamine or Desferal (prescription name) has been used extensively as an iron chelator particularly in protection against iron-induced oxidative stress^38^,^39^. Apart from its antioxidant activity, deferoxamine has been shown to be cytotoxic to a number of human cancer cells and tumors, mainly by altering signaling pathways which are crucial for cell proliferation^40^,^41^.

The presence of these biologically active compounds in MUSC 136^T^ may account for the observed reducing power in antioxidant assays as well as its cytotoxic activity in the various cancer cells. Principally, by inhibiting oxidative stress induced mutagenesis and signaling cascades these compounds might prevent or treat cancer. The results obtained in the current study demonstrate that MUSC 136^T^ extract has tremendous potential for development of cancer preventive agent, meriting further downstream mechanistic studies.

### Description of *Streptomyces malaysiense* sp. nov

*Streptomyces malaysiense* (mal.ay.si.en’se. N.L. neur. adj. malaysiense belonging/pertaining to Malaysia, the source of the soil from which the organism was isolated).

Cells stain Gram-positive and form yellowish white aerial and pale yellow substrate mycelium on ISP 2 medium. The colors of the aerial and substrate mycelium are media-dependent (See Supplementary Table S1).

Grows well on ISP 2, ISP 5, ISP 6, ISP 7 agar, actinomycetes isolation agar, nutrient agar and starch casein agar after 7–14 days at 28 °C; grows moderately on *Streptomyces* agar and ISP 3 agar, and does not grow on ISP 4 agar. Grows at 26–40 °C (optimum 28–32 °C), pH 6.0–7.0 (optimum pH 7.0), with 0–6% NaCl tolerance (optimum 0–2%). Cells are positive for catalase but negative for hemolytic activity and melanoid pigment production. Soluble starch, tributyrin (lipase) and casein are hydrolysed but carboxymethylcellulose, chitin and xylan are not. The following compounds are utilized as sole carbon sources: acetic acid, acetoacetic acid, α-D-glucose, α-hydroxy-butyric acid, α-keto-butyric acid, α-keto-glutaric acid, β-hydroxyl-D,L-butyric acid, bromo-succinic acid, citric acid, D-arabitol, D-aspartic acid, D-cellobiose, dextrin, D-fructose, D-fructose-6-phosphate, D-fucose, D-galactose, D-galacturonic acid, D-glucose-6-phosphate, D-gluconic acid, D-glucuronic acid, D-lactic acid methyl ester, D-malic acid, D-maltose, D-mannitol, D-mannose, D-raffinose, D-saccharic acid, D-trehalose, D-turanose, formic acid, gelatin, gentiobiose, glucuronamide, glycerol, glycyl-L-proline, inosine, L-fucose, L-galactonic acid lactone, L-lactic acid, L-malic acid, L-rhamnose, methyl pyruvate, mucic acid, N-acetyl-β-D-mannosamine, N-acetyl-D-galactosamine, N-acetyl-D-glucosamine, N-acetyl-neuraminic acid, pectin, p-hydroxyl-phenylacetic acid, propionic acid, quinic acid, stachyose, sucrose, Tween 40 and γ-amino-butyric acid. The following compounds are not utilized as sole carbon sources: α-D-lactose, β-methyl-D-glucoside, D-melibiose, D-salicin, D-serine, D-sorbitol, 3-methyl glucose and myo-inositol. L-alanine, L-arginine, L-aspartic acid, L-glutamic acid, L-histidine, L-pyroglutamic acid and L-serine are utilized as sole nitrogen sources. Cells are sensitive to ampicillin sulbactam, cefotaxime, chloramphenicol, erythromycin, gentamicin, penicillin G, tetracycline and vancomycin; resistant to ampicillin and nalidixic acid. The cell wall peptidoglycan contains LL-diaminopimelic acid. The cell wall sugars are glucose and ribose. The predominant menaquinones are MK-9(H_8_) and MK-9(H_6_). The polar lipids consist of aminopilid, diphosphatidylglycerol, glycolipid, lipid, phosphatidylethanolamine, phosphatidylinositol, phospholipid and phosphoglycolipid. The major cellular fatty acids are anteiso-C_15: 0_, iso-C_16: 0_, iso-C_15: 0_ and anteiso-C_17: 0_.

The type strain is MUSC 136^T^ (=DSM 100712^T^ = MCCC 1K01246^T^), isolated from mangrove soil collected from the Tanjung Lumpur mangrove forest located in the state of Pahang, Peninsular Malaysia. The 16S rRNA gene sequence of strain MUSC 136^T^ has been deposited in GenBank/EMBL/DDBJ under the accession number KJ632663. The G+C content of the genomic DNA of the type strain is 72.3 mol%.

## Materials and Methods

### Isolation and maintenance of isolate

Strain MUSC 136^T^ was isolated from a soil sample collected at site MUSC-TLS3 (3° 48′ 11.1″ N 103 ° 26′ 6.9″ E), located in the mangrove forest of Tanjung Lumpur in the state of Pahang, Peninsular Malaysia in December 2012. Topsoil samples of the upper 20 cm layer (after removing the top 2–3 cm) were collected and sampled into sterile plastic bags using an aseptic metal trowel, and stored at −20 °C. Air-dried soil samples were ground with a mortar and pestle followed by selective pretreatment using wet heat in sterilized water^16^. The pretreated air-dried soil (5 g) in 45 mL sterilized water was spread onto the isolation medium ISP 2 supplemented with cycloheximide (25 μg/mL) and nystatin (10 μg/mL), and incubated at 28 °C for 14 days. Pure cultures of strain MUSC 136^T^ were isolated and maintained on ISP 2 medium slants at 28 °C and preserved as glycerol suspensions (20%, v/v) at −20 °C.

### Genomic and phylogenetic analyses

DNA extraction for PCR was performed as described previously^12^ while 16S rRNA gene was amplified as described by Lee *et al.*^15^. The 16S rRNA gene sequence of strain MUSC 136^T^ was aligned with representative sequences of related type strains of the genus *Streptomyces* retrieved from the GenBank/EMBL/DDBJ databases. Phylogenetic trees were constructed with the neighbour-joining (Fig. 2) and maximum-likelihood algorithms (See Supplementary Fig. S1). Evolutionary distances for the neighbour-joining algorithm were computed using Kimura’s two-parameter model^42^. The EzTaxon-e server (http://eztaxon-e.ezbiocloud.net/) was used for calculations of sequence similarity^43^.

BOX-PCR fingerprint analysis and DDH were performed as previously described^44^,^45^. The extraction of genomic DNA for DNA-DNA hybridization of strain MUSC 136^T^, *Streptomyces misionensis* NBRC 13063^T^, *Streptomyces phaeoluteichromatogenes* DSM 41898^T^ and *Streptomyces rutgersensis* NBRC 12819^T^ were carried out by the Identification Service of the DSMZ, Braunschweig, Germany. The G+C content of strain MUSC 136^T^ was determined by HPLC^46^.

### Phenotypic characteristics

Cultural characteristics of strain MUSC 136^T^ were determined following growth on ISP 2, ISP 3, ISP 4, ISP 5, ISP 6 and ISP 7 agar, actinomycetes isolation agar (AIA), *Streptomyces* agar (SA), starch casein agar (SCA) and nutrient agar for 14 days at 28 °C. Light microscopy (80i, Nikon) and scanning electron microscopy (JEOL-JSM 6400) were used to observe the morphology of the strain after incubation on ISP 2 medium at 28 °C for 7–14 days. The designation of colony color was determined using the ISCC-NBS color charts while gram staining performed by standard Gram reaction and confirmed by KOH lysis^15^,^16^,^17^. The effects of pH and salinity on growth of MUSC 136^T^ were evaluated in tryptic soy broth (TSB), while effects of temperature were studied in ISP 2 agar^15^. Production of melanoid pigments were examined using ISP 7 agar while its hemolytic activity was assessed on blood agar medium containing 5% (w/v) peptone, 3% (w/v) yeast extract, 5% (w/v) NaCl and 5% (v/v) horse blood after incubation at 32 °C for 7–14 days^16^. Amylolytic, cellulase, chitinase, lipase, protease, xylanase and catalase activities were determined on ISP 2 following protocols described by Lee *et al.*^47^. Antibiotic susceptibility tests were performed by the disc diffusion method as described by Shieh *et al.*^48^ Carbon-source utilization and chemical sensitivity assays were determined using Biolog GenIII MicroPlates (Biolog, USA) according to the manufacturer’s instructions.

All phenotypic assays mentioned above were performed concurrently for strains MUSC 136^T^, *Streptomyces misionensis* NBRC 13063^T^, *Streptomyces phaeoluteichromatogenes* DSM 41898^T^ and *Streptomyces rutgersensis* NBRC 12819^T^.

### Chemotaxonomic characteristics

The analyses of peptidoglycan amino acid composition and sugars of strain MUSC 136^T^ were carried out by the Identification Service of the DSMZ^15^,^16^,^17^. Analysis of respiratory quinones, polar lipids and fatty acids were carried out by the Identification Service of the DSMZ. Major diagnostic cell wall sugars of strain MUSC 136^T^ were obtained as described by Whiton *et al.*^49^ and analyzed by TLC on cellulose plates^50^.

### Extract preparation of MUSC 136^T^

MUSC 136^T^ was grown in TSB as seed medium for 14 days prior to fermentation process. The fermentation medium, FM3 was autoclaved at 121 °C for 15 min prior to experiment^12^,^51^. Fermentation was carried out in 200 mL FM3, shaking at 200 rpm for 7–10 days at 28 °C, inoculated with 200 μL seed media^16^,^17^. The resulting FM3 medium was recovered by centrifugation at 12000 ***g*** for 15 min. The supernatant was filtered and subjected to freeze drying process. The freeze-dried sample was extracted with methanol and the final extract concentrated by rotary evaporator at 40 °C. The final concentrate was suspended in DMSO prior to bioactivity screening assays.

### Evaluation of antioxidant activity using different assays

2,2-diphenyl-1-picrylhydrazyl (DPPH) was used to examine antioxidant activity by measuring its hydrogen donating or radical scavenging ability. Scavenging activity against DPPH free radicals by MUSC 136^T^ extract was determined using previously described protocol^17^,^52^. The reduction in radical content was measured by the decrease in the absorbance at 515 nm. 2,2′-azino-bis (3-ethylbenzothiazoline-6-sulphonic acid) (ABTS) assay was performed as previously described in published literature with some modifications^53^. ABTS radical cation (ABTS∙) was generated by reacting ABTS stock solution (7 mM) and potassium persulphate (2.45 mM) for 24 hour prior to assay. The change in radical amount was indicated by decrease in absorbance at 743 nm. Superoxide anion scavenging activity was determined using commercially available colorimetric microtiter plate method (19160 SOD Assay Kit-WST, Sigma Aldrich) according to manufacturer’s protocol. Metal chelating activity was determined by measuring formation of Fe^2+^-ferrozine complex as previously described by Manivasagan *et al.* with slight modification^54^. 2 mM of FeSO_4_ was added to extract and the reaction was initiated by adding 5 mM of ferrozine before measuring at 562 nm using spectrophotometer.

### Cell lines maintenance and growth condition

All human derived cancer cell lines used in the study were maintained in RPMi 1640 supplemented with 10% FBS in a humidified incubator (5% CO_2_ in air at 37 °C)^55^. The cancer cell lines included were cervical cancer cell, Ca Ski; colon cancer cell, HCT-116 and HT-29; lung carcinoma cell line, A549.

### Cytotoxicity activity determination using 3-(4,5-dimethylthazol-2yl)-2,5-diphenyl tetrazolium-bromide (MTT) assay

The cytotoxic activity of MUSC 136^T^ extract was investigated using 3-(4,5-dimethylthazol-2yl)-2,5-diphenyl tetrazolium-bromide (MTT) assay according to previously established method^18^. The determination of cell viability was performed using a microplate reader at 570 nm (with 650 nm as reference wavelength).

### Measurement of intracellular GSH content

Intracellular GSH content was measured using CellMetrix™ Intracellular GSH Assay Kit according to manufacturer’s protocol. Cells were seeded in 6-well dishes at density of 3 × 10^5^ and allowed to adhere overnight. Subsequently, the cells were treated with MUSC 136^T^ extract. After the incubation period, cells were harvested and stained prior to analysis using BD FACSVerse^TM^ flow cytometer.

### Measurement of p53 protein

The status of p53 protein was investigated with flow cytometry system as described by Goh *et al.* with slight modification^24^. Cells were seeded in 6-well dishes at density of 3 × 10^5^ and allowed to adhere overnight before treated with MUSC 136^T^ extract. After the incubation period, cells were then washed twice with PBS, fixed with 4% paraformaldehyde with 0.5% sodium azide before permeabilized with Perm/Wash™ buffer (BD Biosciences). For detection of p53, the cells were incubated with 100 μL of Perm/Wash™ buffer containing mouse anti-human p53 monoclonal IgG_1_ or IgG_1_ isotype control. After washing procedure, the cells were further incubated with FITC-conjugated goat anti-mouse IgG_1_ at 4 °C in the dark for 30 min. Finally, the cells were then washed with Perm/Wash™ buffer before analysis by using BD FACSVerse^TM^ flow cytometer.

### Gas chromatography-mass spectrometry (GC-MS) analysis

GC-MS analysis was performed as previously described^17^. Analysis of MUSC 136^T^ extract was performed on an Agilent Technologies 6980N (GC) equipped with 5979 Mass Selective Detector (MS) using a HP-5MS (5% phenyl methyl siloxane) capillary column of dimensions 30.0 m × 250 μm × 0.25 μm and helium as carrier gas at 1 mL/min. The column temperature was programmed initially at 40 °C for 10 min, followed by an increase of 3 °C/min to 250 °C and was kept isothermally for 5 min. The MS was operating at 70 eV. The constituents were identified by comparison of their mass spectral data with those from NIST 05 Spectral Library.

### Statistical analysis

Experiments to investigate antioxidant and cytotoxic activities were done in quadruplicate while measurement of p53 and GSH using flow cytometry in triplicate. Data analysis was performed with SPSS statistical analysis software and the results were expressed as mean ± standard deviation (SD). One-way analysis of variance (ANOVA) followed by the appropriate post hoc test (Tukey) was used to establish whether significant differences existed between groups. A difference was considered statistically significant when *p* ≤ 0.05.

## Additional Information

**How to cite this article**: Ser, H.-L. *et al.* *Streptomyces malaysiense* sp. nov.: A novel Malaysian mangrove soil actinobacterium with antioxidative activity and cytotoxic potential against human cancer cell lines. *Sci. Rep.* **6**, 24247; doi: 10.1038/srep24247 (2016).

## Supplementary Material

Supplementary Information

## Figures and Tables

**Table 1:** Differentiation characteristics of strain MUSC 136^T^ and type strains of phylogenetically closely related species of the genus *Streptomyces.*

Characteristic	1	2	3	4
*Morphology (on ISP 2):*				
Color of aerial mycelium	Yellow white	Yellowish white	Yellowish white	Light yellow
Color of substrate mycelium	Pale yellow	Strong yellow	Yellowish gray	Brilliant greenish yellow
*Growth at:*				
24 °C	(+)	(+)	(+)	(+)
40 °C	(+)	+	+	−
pH 6	(+)	(+)	(+)	+
4% NaCl	(+)	+	−	(+)
Catalase	+	+	+	+
Hemolytic	−	−	−	−
*Hydrolysis of:*				
Casein (protease)	+	−	+	−
Tributyrin (lipase)	+	+	+	−
Starch (amylolytic)	+	+	+	+
Carboxymethylcellulose (cellulase)	−	+	+	+
Xylan (xylanase)	−	+	−	−
*Carbon source utilization:*				
Sucrose	+	+	+	−
Stachyose	+	+	+	−
D-raffinose	+	+	−	−
α-D-lactose	−	+	+	+
D-melibiose	−	+	−	−
β-methyl-D-glucoside	−	−	+	−
D-salicin	−	−	+	−
N-acetyl-b-D-mannosamine	+	−	+	−
N-acetyl-neuraminic acid	+	−	+	+
D-fucose	+	+	+	−
L-fucose	+	+	+	−
L-rhamnose	−	−	+	−
D-sorbitol	−	+	+	−
Myo-inositol	−	+	+	−
D-glucose-6-PO_4_	+	+	+	−
D-aspartic acid	+	+	+	−
D-serine	−	+	+	−
Chemical sensitivity assays:				
Troleandomycin	+	+	+	−
Vancomycin	+	+	+	−
Fusidic acid	+	+	+	−
Rifamycin RV	+	+	+	−
Minocycline	+	+	+	−
Lincomycin	+	+	+	−
Niaproof 4	+	+	+	−

Strains: 1, *Streptomyces malaysiense* sp. nov. MUSC 136^T^; 2, *Streptomyces misionensis* NBRC 13063^T^; 3, *Streptomyces phaeoluteichromatogenes* DSM 41898^T^; 4, *Streptomyces rutgersensis* NBRC 12819^T^. All data were obtained concurrently in this study. +, Positive; −, negative; (+), weak.

All strains are positive for utilization of Dextrin, D-maltose, D-trehalose, D-cellobiose, Gentiobiose, D-turanose, N-acetyl-D-glucosamine, N-acetyl-D-galactosamine, α-D-glucose, D-mannose, D-fructose, D-galactose, Inosine, D-mannitol, D-arabitol, Glycerol, D-fructose-6-PO_4_, Gelatin, Glycyl-L-proline, L-alanine, L-arginine, L-aspartic acid, L-glutamic acid, L-histidine, L-serine, Pectin, D-galacturonic acid, L-galactonic acid lactone, D-gluconic acid, D-glucuronic acid, Glucuronamide, Methyl pyruvate, L-lactic acid, L-malic acid, Bromo-succinic acid, Tween 40, γ-amino-butyric acid, α-hydroxy-butyric acid, β-hydroxy-D,L-butyric acid, α-keto-butyric acid, Acetoacetic acid, Propionic acid and Acetic acid. All strains are negative for assimilation of 3-methyl glucose.

**Table 2:** Cellular fatty acid composition of strain MUSC 136^T^ and its closely related *Streptomyces* species.

Fatty acid (%)	1	2	3	4
iso-C_13:0_	0.2	0.1	0.2	0.2
anteiso-C_13:0_	0.3	0.3	–	0.2
iso-C_14:0_	2.5	1.8	4.8	2.8
C_14:0_	0.6	0.2	0.2	0.6
iso-C_15:0_	12.2	7.2	12.3	5.2
anteiso-C_15:0_	35.3	40.1	35.5	32.2
C_15:1_ B	0.2	–	–	0.5
C_15:0_	2.9	0.7	1.6	5.5
iso-C_16:1_ H	1.8	1.6	1.3	1.0
iso-C_16:0_	12.4	14.4	17.7	10.3
C_16:1_ Cis 9	3.9	1.3	0.7	3.7
anteiso-C_15:0_ 2OH	–	–	–	2.1
C_16:0_	6.5	4.0	3.4	12.4
anteiso-C_17:1_ C	4.3	4.1	2.8	2.2
iso-C_17:0_	2.4	2.4	3.5	2.2
anteiso-C_17:0_	11.8	19.3	13.4	13.9
C_17:1_ Cis 9	0.3	0.1	0.2	1.3
C_17:0_ CYCLO	0.1	0.4	0.3	0.4
C_17:0_	0.4	0.2	0.2	2.4

Strains: 1, *Streptomyces malaysiense* sp. nov. MUSC 136^T^; 2, *Streptomyces misionensis* NBRC 13063^T^; 3, *Streptomyces phaeoluteichromatogenes* DSM 41898^T^; 4, *Streptomyces rutgersensis* NBRC 12819^T^. –, <0.1% or not detected. All data are obtained concurrently from this study.

**Table 3:** Radical scavenging activity of MUSC 136^T^ evaluated using DPPH, ABTS, metal chelating and superoxide dismutase (SOD)-like assays (−, not available; *, *p *> 0.05).

	Conc. (mg/mL)	Mean ± standard error (%)			
		DPPH	ABTS	SOD	Metal-chelating
MUSC 136^T^	0.25	4.87 ± 0.71	7.51 ± 2.19*	45.98 ± 2.81*	10.22 ± 1.58*
	0.50	10.26 ± 2.44	11.59 ± 1.50*	56.93 ± 3.76*	17.00 ± 3.73*
	1.00	11.15 ± 3.26	15.95 ± 2.34*	59.72 ± 6.19*	22.97 ± 1.51*
	2.00	27.24 ± 1.91*	27.87 ± 2.19*	68.27 ± 3.67*	37.01 ± 2.59*
Gallic acid	0.0625	64.90 ± 0.36*	42.61 ± 1.78*	−	−
Ascorbic acid	0.0078125	−	−	40.45 ± 2.16*	−
EDTA	0.025	−	−	−	43.25 ± 2.70*

**Table 4:** Compounds identified from MUSC 136^T^ extract using GC-MS.

No	Retention time (min)	Compound	Formula	Molecular weight	Quality
1	14.370	Isomeric dihydro-methyl-furanone	C_5_H_6_O_2_	98	90
2	39.484	1-Pentadecene	C_15_H_30_	210	83
3	44.502	Phenol, 2,5-bis (1,1-dimethylethyl)-	C_14_H_22_O	206	90
4	51.569	(3R,8aS)-3-methyl-1,2,3,4,6,7,8,8a-octahydropyrrolo[1,2-a]pyrazine-1,4-dione	C_8_H_12_N_2_O_2_	168	90
5	53.074	1,4-diaza-2,5-dioxobicyclo[4.3.0]nonane	C_7_H_10_N_2_O_2_	154	94
6	54.956	Tetradecanoic acid, 12-methyl-, methyl ester	C_16_H_32_O_2_	256	64
7	55.220	Pyrrolo[1,2-a]pyrazine-1,4-dione, hexahydro-3-(2-methylpropyl)-	C_11_H_18_N_2_O_2_	210	64
8	58.063	Pentadecanoic acid, 14-methyl-, methyl ester	C_17_H_34_O_2_	270	91
9	71.544	Deferoxamine	C_25_H_48_N_6_O_8_	560	47

## References

[b1] Jemal A. *et al.* Global cancer statistics. CA Cancer J. Clin. 61, 69–90, 10.3322/caac.20107 (2011). 21296855

[b2] Jemal A., Center M. M., DeSantis C. & Ward E. M. Global patterns of cancer incidence and mortality rates and trends. Cancer Epidemiol. Biomarkers Prev. 19, 1893–1907, 10.1158/1055-9965.EPI-10-0437 (2010). 20647400

[b3] Chin Y.-W., Balunas M. J., Chai H. B. & Kinghorn A. D. Drug discovery from natural sources. AAPS J. 8, E239–E253. 10.1007/BF02854894 (2006). 16796374 PMC3231566

[b4] Lalla R. V., Saunders D. P. & Peterson D. E. Chemotherapy or radiation-induced oral mucositis. Dent. Clin. North Am. 58, 341–349, 10.1016/j.cden.2013.12.005 (2014). 24655526

[b5] Robles-Fernandez I. *et al.* Antitumor properties of natural compounds and related molecules. Recent Pat. Anticancer Drug Discov. 8, 203–215 (2013). 23157341 10.2174/1574891x113089990034

[b6] Newman D. J. & Cragg G. M. Marine-sourced anti-cancer and cancer pain control agents in clinical and late preclinical development. Mar. Drugs 12, 255–278, 10.3390/md12010255 (2014). 24424355 PMC3917273

[b7] Berdy J. Bioactive microbial metabolites. J. Antibiotics 58, 1–26, 10.1038/ja.2005.1 (2005). 15813176

[b8] Demain A. L. & Sanchez S. Microbial drug discovery: 80 years of progress. J. Antibiotics 62, 5–16, 10.1038/ja.2008.16 (2009). 19132062 PMC7094699

[b9] Waksman S. A. & Henrici A. T. The nomenclature and classification of the actinomycetes. J. Bacteriol. 46, 337 (1943). 16560709 10.1128/jb.46.4.337-341.1943 PMC373826

[b10] Alongi D. M. Mangrove forests: resilience, protection from tsunamis, and responses to global climate change. Estuar.Coast. Shelf Sci. 76, 1–13, 10.1016/j.ecss.2007.08.024 (2008).

[b11] Lee L. H. *et al.* Diversity and antimicrobial activities of actinobacteria isolated from tropical mangrove sediments in Malaysia. Scientific World J. 2014, 698178, 10.1155/2014/698178 (2014). PMC4138949 25162061

[b12] Hong K. *et al.* Actinomycetes for marine drug discovery isolated from mangrove soils and plants in China. Mar. drugs 7, 24–44, 10.3390/md7010024 (2009). 19370169 PMC2666887

[b13] Azman A. S., Othman I., Velu S. S., Chan K. G. & Lee L. H. Mangrove rare actinobacteria: taxonomy, natural compound, and discovery of bioactivity. Front. Microbiol. 6, 856, 10.3389/fmicb.2015.00856 (2015). 26347734 PMC4542535

[b14] Sui J.-L. *et al.* *Streptomyces sanyensis* sp. nov., isolated from mangrove sediment. Int. J. Syst. Evol. Microbiol. 61, 1632–1637, 10.1099/ijs.0.023515-0 (2011). 20693357

[b15] Lee L. H. *et al.* *Streptomyces pluripotens* sp. nov., a bacteriocin-producing streptomycete that inhibits meticillin-resistant *Staphylococcus aureus*. Int. J. Syst. Evol. Microbiol. 64, 3297–3306, 10.1099/ijs.0.065045-0 (2014). 24994773

[b16] Ser H.-L. *et al.* *Streptomyces gilvigriseus* sp. nov., a novel actinobacterium isolated from mangrove forest soil. Antonie van Leeuwenhoek 107, 1369–1378, 10.1007/s10482-015-0431-5 (2015). 25863667

[b17] Ser H.-L. *et al.* Presence of antioxidative agent, Pyrrolo[1,2-a]pyrazine-1,4-dione, hexahydro- in newly isolated *Streptomyces mangrovisoli* sp. nov. Front. Microbiol. 6, 854, 10.3389/fmicb.2015.00854 (2015). 26347733 PMC4542459

[b18] Williams S. T., Goodfellow M. & Alderson G. Genus *Streptomyces* Waksman and Henrici 1943, 339^AL^. In Bergey’s Manual of Systematic Bacteriology, Volume 4. (eds Williams S. T. *et al.* ), 2452–2492, (Williams & Wilkins, 1989).

[b19] Wayne L. G. *et al.* Report of the Ad Hoc Committee on Reconciliation of Approaches to Bacterial Systematics. Int. J. Syst. Evol. Microbiol. 37, 463–464, 10.1099/00207713-37-4-463 (1987).

[b20] Lechevalier M. P. & Lechevalier H. Chemical composition as a criterion in the classification of aerobic actinomycetes. Int. J. Syst. Bacteriol. 20, 435–443, 10.1099/00207713-20-4-435 (1970).

[b21] Kim S. B., Lonsdale J., Seong C. N. & Goodfellow M. *Streptacidiphilus* gen. nov., acidophilic actinomycetes with wall chemotype I and emendation of the family *Streptomycetaceae* (Waksman and Henrici (1943)^AL^) emend. Rainey *et al.* 1997. Antonie van Leeuwenhoek 83, 107–116 (2003). 12785304 10.1023/a:1023397724023

[b22] Reuter S., Gupta S. C., Chaturvedi M. M. & Aggarwal B. B. Oxidative stress, inflammation, and cancer: how are they linked? Free Rad. Biol. Med. 49, 1603–1616, 10.1016/j.freeradbiomed.2010.09.006 (2010). 20840865 PMC2990475

[b23] Kawanishi S., Oikawa S. & Murata M. Evaluation for safety of antioxidant chemopreventive agents. Antioxid. Redox Signal 7, 1728–1739, 10.1089/ars.2005.7.1728 (2005). 16356133

[b24] Goh B. H., Chan C. K., Kamarudin M. N. & Abdul Kadir H. *Swietenia macrophylla* King induces mitochondrial-mediated apoptosis through p53 upregulation in HCT116 colorectal carcinoma cells. J. Ethnopharmacol. 153, 375–385, 10.1016/j.jep.2014.02.036 (2014). 24613274

[b25] Schneider-Stock R. *et al.* 5-Aza-cytidine is a potent inhibitor of DNA methyltransferase 3a and induces apoptosis in HCT-116 colon cancer cells via Gadd45- and p53-dependent mechanisms. J. Pharmacol. Exp. Ther. 312, 525–536, 10.1124/jpet.104.074195 (2005). 15547111

[b26] Makizumi R., Yang W.-L., Owen R. P., Sharma R. R. & Ravikumar T. S. Alteration of drug sensitivity in human colon cancer cells after exposure to heat: implications for liver metastasis therapy using RFA and chemotherapy. Int. J. Clin. Exp. Med. 1, 117–129 (2008). 19079666 PMC2596320

[b27] Lin-Lee Y.-C., Tatebe S., Savaraj N., Ishikawa T. & Tien Kuo M. Differential sensitivities of the MRP gene family and γ-glutamylcysteine synthetase to prooxidants in human colorectal carcinoma cell lines with different p53 status. Biochem. Pharmacol. 61, 555–563, 10.1016/S0006-2952(00)00592-X (2001). 11239498

[b28] Shukla S. & Gupta S. Apigenin-induced prostate cancer cell death is initiated by reactive oxygen species and p53 activation. Free Rad. Biol. Med. 44, 1833–1845, 10.1016/j.freeradbiomed.2008.02.007 (2008). 18342637 PMC2538676

[b29] Jog R., Pandya M., Nareshkumar G. & Rajkumar S. Mechanism of phosphate solubilization and antifungal activity of *Streptomyces* spp. isolated from wheat roots and rhizosphere and their application in improving plant growth. Microbiol. 160, 778–788, 10.1099/mic.0.074146-0 (2014). 24430493

[b30] Olano C., Mendez C. & Salas J. A. Antitumor compounds from actinomycetes: from gene clusters to new derivatives by combinatorial biosynthesis. Nat. Prod. Rep. 26, 628–660, 10.1039/b822528a (2009). 19387499

[b31] Wang C. *et al.* Antifungal activity of volatile organic compounds from *Streptomyces alboflavus* TD-1. FEMS Microbiol. Lett. 341, 45–51, 10.1111/1574-6968.12088 (2013). 23351181

[b32] Narendhran S., Rajiv P., Vanathi P. & Sivaraj R. Spectroscopic analysis of bioactive compounds from *Streptomyces cavouresis* kuv39: Evaluation of antioxidant and cytotoxicity activity. Int. J. Pharm. Pharmaceut. Sci. 6, 319–322 (2014).

[b33] Gopi M., Dhayanithi N. B., Devi K. N. & Kumar T. T. A. Marine natural product, Pyrrolo [1,2-a] pyrazine-1,4-dione, hexahydro-(C_7_H_10_N_2_O_2_) of antioxidant properties from *Bacillus* species at Lakshadweep archipelago. J. Coast. Life Med. 2, 632–637, 10.12980/JCLM.2.201414J40 (2014).

[b34] Wojcik M., Burzynska-Pedziwiatr I. & Wozniak L. A. A review of natural and synthetic antioxidants important for health and longevity. Curr. Med. Chem. 17, 3262–3288, 10.2174/092986710792231950 (2010). 20666718

[b35] Barona-Gomez F., Wong U., Giannakopulos A. E., Derrick P. J. & Challis G. L. Identification of a cluster of genes that directs desferrioxamine biosynthesis in *Streptomyces coelicolor* M145. J. Am. Chem. Soc. 126, 16282–16283, 10.1021/ja045774k (2004). 15600304

[b36] Yamanaka K. *et al.* Desferrioxamine E produced by *Streptomyces griseus* stimulates growth and development of *Streptomyces tanashiensis*. Microbiol. 151, 2899–2905, 10.1099/mic.0.28139-0 (2005). 16151202

[b37] WHO. *WHO model list of essential medicines: 19*^*th*^ *list (updated) April 2015*. (2015). Available at: http://www.who.int/medicines/publications/essentialmedicines/en/. (Accessed: 19^th^ August 2015).

[b38] van der Kraaij A. M., Mostert L. J., van Eijk H. G. & Koster J. F. Iron-load increases the susceptibility of rat hearts to oxygen reperfusion damage. Protection by the antioxidant (+)-cyanidanol-3 and deferoxamine. Circulation 78, 442–449, 10.1161/01.CIR.78.2.442 (1988). 3396180

[b39] Lan J. & Jiang D. H. Desferrioxamine and vitamin E protect against iron and MPTP-induced neurodegeneration in mice. J. Neural Transm. 104, 469–481, 10.1007/BF01277665 (1997). 9295179

[b40] Tomoyasu S. *et al.* Suppression of HL-60 cell proliferation by deferoxamine: changes in *c-myc* expression. Anticancer Res. 13, 407–410 (1992). 8517655

[b41] Yamasaki T., Terai S. & Sakaida I. Deferoxamine for advanced hepatocellular carcinoma. New Eng. J. Med. 365, 576–578, 10.1056/NEJMc1105726 (2011). 21830988

[b42] Kimura M. A simple method for estimating evolutionary rates of base substitutions through comparative studies of nucleotide sequences. J. Mol. Evol. 16, 111–120 (1980). 7463489 10.1007/BF01731581

[b43] Kim O. S. *et al.* Introducing EzTaxon-e: a prokaryotic 16S rRNA gene sequence database with phylotypes that represent uncultured species. Int. J. Syst. Evol. Microbiol. 62, 716–721, 10.1099/ijs.0.038075-0 (2012). 22140171

[b44] Lee L. H. *et al.* *Novosphingobium malaysiense* sp. nov. isolated from mangrove sediment. Int. J. Syst. Evol. Microbiol. 64, 1194–1201, 10.1099/ijs.0.059014-0 (2014). 24408529

[b45] Lee L.-H. *et al.* *Microbacterium mangrovi* sp. nov., an amylolytic actinobacterium isolated from mangrove forest soil. Int. J. Syst. Evol. Microbiol. 64, 3513–3519, 10.1099/ijs.0.059014-0 (2014). 25056298

[b46] Mesbah M., Premachandran U. & Whitman W. B. Precise measurement of the G+C content of deoxyribonucleic acid by high-performance liquid chromatography. Int. J. Syst. Bacteriol. 39, 159–167, 10.1099/00207713-39-2-159 (1989).

[b47] Lee L. H. *et al.* *Mumia flava* gen. nov., sp. nov., an actinobacterium of the family Nocardioidaceae. Int. J. Syst. Evol. Microbiol. 64, 1461–1467, 10.1099/ijs.0.058701-0 (2014). 24449791

[b48] Shieh W. Y., Chen Y. W., Chaw S. M. & Chiu H. H. *Vibrio ruber* sp. nov., a red, facultatively anaerobic, marine bacterium isolated from sea water. Int. J. Syst. Evol. Microbiol. 53, 479–484, doi: 10.1099/ijs.0.02307-0 (2003). 12710616

[b49] Whiton R. S., Lau P., Morgan S. L., Gilbart J. & Fox A. Modifications in the alditol acetate method for analysis of muramic acid and other neutral and amino sugars by capillary gas chromatography-mass spectrometry with selected ion monitoring. J. Chromatogr. 347, 109–120, 10.1016/S0021-9673(01)95474-3 (1985). 4086626

[b50] Staneck J. L. & Roberts G. D. Simplified approach to identification of aerobic actinomycetes by thin-layer chromatography. Appl. Microbiol. 28, 226–231 (1974). 4605116 10.1128/am.28.2.226-231.1974 PMC186691

[b51] Lee L.-H. *et al.* Molecular characterization of Antarctic actinobacteria and screening for antimicrobial metabolite production. World J. Microbiol. Biotech. 28, 2125–2137, 10.1007/s11274-012-1018-1 (2012). 22806035

[b52] Al-Henhena N. *et al.* Chemopreventive effects of *Strobilanthes crispus* leaf extract on azoxymethane-induced aberrant crypt foci in rat colon. Sci. Rep. 5, 10.1038/srep13312 (2015). PMC4642519 26307342

[b53] Miser-Salihoglu E., Akaydin G., Caliskan-Can E. & Yardim-Akaydin S. Evalution of antioxidant activity of various herbal folk medicines. J. Nutri. Food Sci. 3, 3–5, 10.4172/2155-9600.1000222 (2013).

[b54] Manivasagan P., Venkatesan J., Sivakumar K. & Kim S. K. Production, characterization and antioxidant potential of protease from *Streptomyces* sp. MAB18 using poultry wastes. BioMed Res. Int. 2013, 496586–496586, 10.1155/2013/496586 (2013). 23991418 PMC3749541

[b55] Tan L. T. H. *et al.* Investigation of antioxidative and anticancer potentials of *Streptomyces* sp. MUM256 isolated from Malaysia mangrove soil. Front. Microbiol. 6, 1316, 10.3389/fmicb.2015.01316 (2015). 26635777 PMC4659911

