# Supplementary material for: Streptomyces malaysiense sp. nov.: A novel Malaysian mangrove soil actinobacterium with antioxidative activity and cytotoxic potential against human cancer cell lines
Source: Sci Rep. 2016 Apr 13;6:24247. doi: 10.1038/srep24247 (PMC4829849; doi:10.1038/srep24247)
Supplement: Supplementary Information [file srep24247-s1.doc]

***Streptomyces malaysiense* sp. nov.: A novel Malaysian mangrove soil actinobacterium with antioxidative activity and cytotoxic potential against human cancer cell lines**

**Hooi-Leng Ser^1^, Uma Devi Palanisamy^1^, Wai-Fong Yin^2^, Kok-Gan Chan^2^, Bey-Hing Goh^1*^, Learn-Han Lee^1*^**

^1^Biomedical Research Laboratory, Jeffrey Cheah School of Medicine and Health Sciences, Monash University Malaysia, 46150 Bandar Sunway, Selangor Darul Ehsan, Malaysia

^2^Division of Genetics and Molecular Biology, Institute of Biological Sciences, Faculty of Science, University of Malaya, 50603 Kuala Lumpur, Malaysia

*** Correspondence:** Learn-Han Lee, Jeffrey Cheah School of Medicine and Health Sciences, Monash University Malaysia, 46150 Bandar Sunway, Selangor Darul Ehsan, Malaysia. lee.learn.han@monash.edu; leelearnhan@yahoo.com; Bey-Hing Goh, Jeffrey Cheah School of Medicine and Health Sciences, Monash University Malaysia, 46150 Bandar Sunway, Selangor Darul Ehsan, Malaysia. goh.bey.hing@monash.edu

**Fig. S1.** Maximum-likelihood tree based on almost complete 16S rRNA sequences (1489 nucleotides) showing relationship between strain MUSC 136^T^ and representatives of some other related taxa. Bootstrap values (>50%) based on 1000 re-sampled datasets are shown at branch nodes. Bar, 0.002 substitutions per site. Asterisks indicate that the corresponding nodes were also recovered using maximum-likelihood tree-making algorithms.

*Streptomyces althioticus* NRRL B-3981^T^ (AY999791)

*Streptomyces griseoincarnatus* LMG 19316^T^ (AJ781321)

*Streptomyces griseoflavus* LMG 19344^T^ (AJ781322)

*Streptomyces malachitofuscus* NBRC 13059^T^ (AB184282)

*Streptomyces tendae* ATCC 19812^T^ (D63873)

*Streptomyces violaceorubidus* LMG 20319^T^ (AJ781374)

*Streptomyces paradoxus* NBRC 14887^T^ (AB184628)

*Streptomyces glaucescens* NBRC 12774^T^ (AB184843)

*Streptomyces viridochromogenes* NBRC 3113^T^ (AB184728)

*Streptomyces thinghirensis* DSM 41919^T^ (FM202482)

*Streptomyces ambofaciens* ATCC 23877^T^ (M27245)

*Streptomyces flaveolus* NBRC 3715^T^ (AB184786)

*Streptomyces nodosus* ATCC 14899^T^ (AF114036)

*Streptomyces nogalater* JCM 4799^T^ (AB045886)

*Streptomyces lavenduligriseus* NBRC 13405^T^ (AB184382)

*Streptomyces eurythermus* ATCC 14975^T^ (D63870)

***Streptomyces* *malaysiense* MUSC136^T^ (KJ632663)**

*Streptomyces phaeoluteichromatogenes* NRRL 5799^T^ (AJ391814)

*Streptomyces misionensis* NBRC 13063^T^ (AB184285)

*Streptomyces aurantiogriseus* NBRC 12842^T^ (AB184188)

*Streptomyces levis* NBRC 15423^T^ (AB184670)

*Streptomyces carpinensis* NBRC 14214^T^ (AB184574)

*Streptomyces intermedius* NBRC 13049^T^ (AB184277)

*Streptomyces aureoverticillatus* NRRL B-3326^T^ (AY999774)

*Streptomyces rutgersensis* NBRC 12819^T^ (AB184170)

*Streptomyces gougerotii* NBRC 3198^T^ (AB184742)

*Streptomyces* *diastaticus* subsp. *diastaticus* NBRC 3714^T^ (AB184785)

**89***

**83***

**87***

**70***

**61***

**96***

**53***

**0.002**

*****

*****

*****

*****

**Fig. S2.** BOX-PCR comparison of strain MUSC 136^T^ and the closest related type strains. Lanes: 1, *Streptomyces malaysiense* sp. nov. MUSC 136^T^; 2, *Streptomyces misionensis* NBRC 13063^T^; 3, *Streptomyces phaeoluteichromatogenes* DSM 41898^T^; 4, *Streptomyces rutgersensis* NBRC 12819^T^; M, GeneRuler 1kb DNA ladder marker.


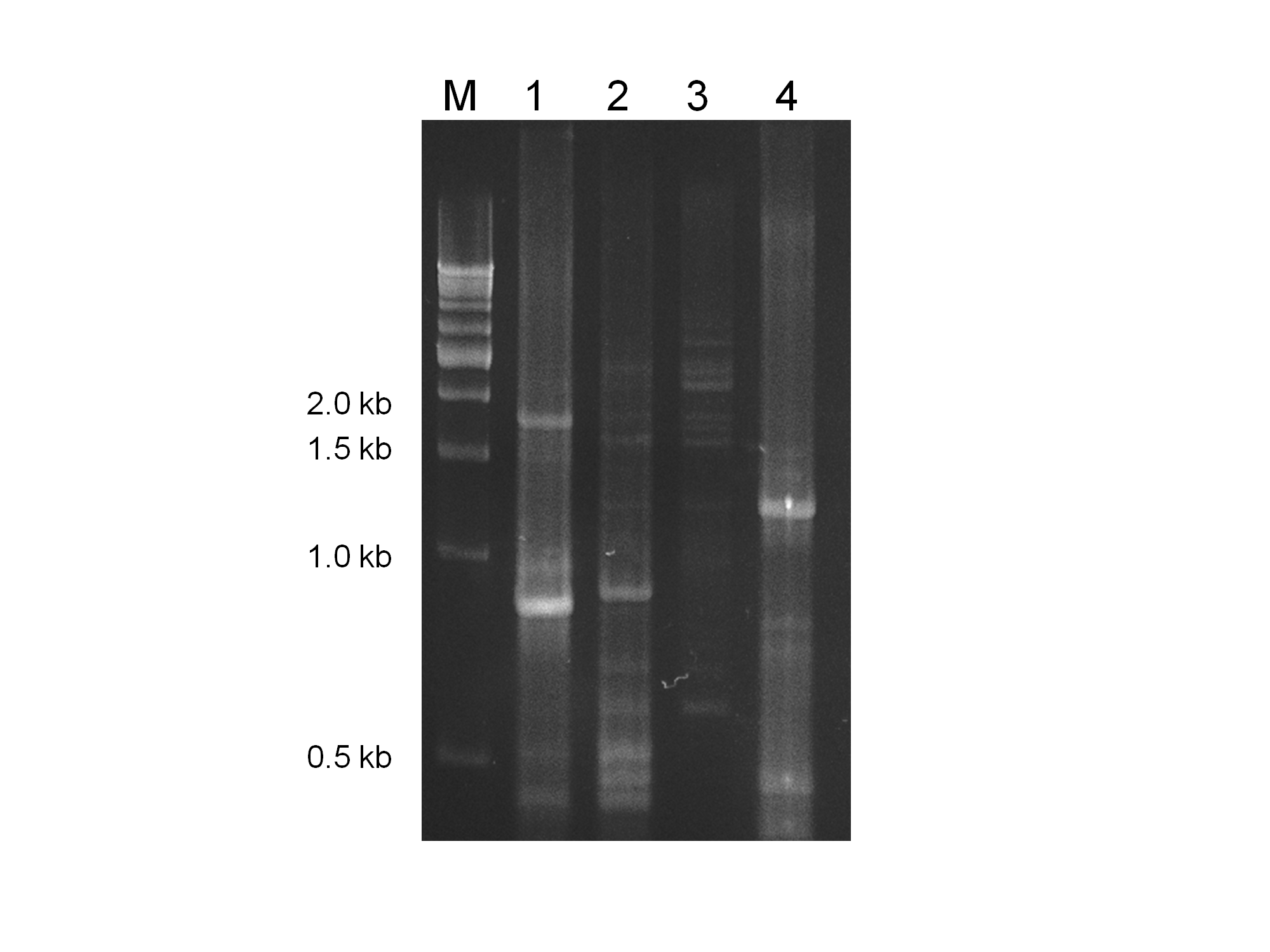


**Fig. S3.** Two dimensional total lipid profile of strain MUSC 136^T^ and *Streptomyces misionensis* NBRC 13063^T^. AL, Aminolipid; DPG, Diphosphatidylglycerol; GL, Glycolipid; PL, Phospholipid; PI, Phosphatidylinositol; PE, Phosphatidylethanolamine; PG, Phosphatidylglycerol; PGL, Phosphoglycolipid; L, Lipid.

**
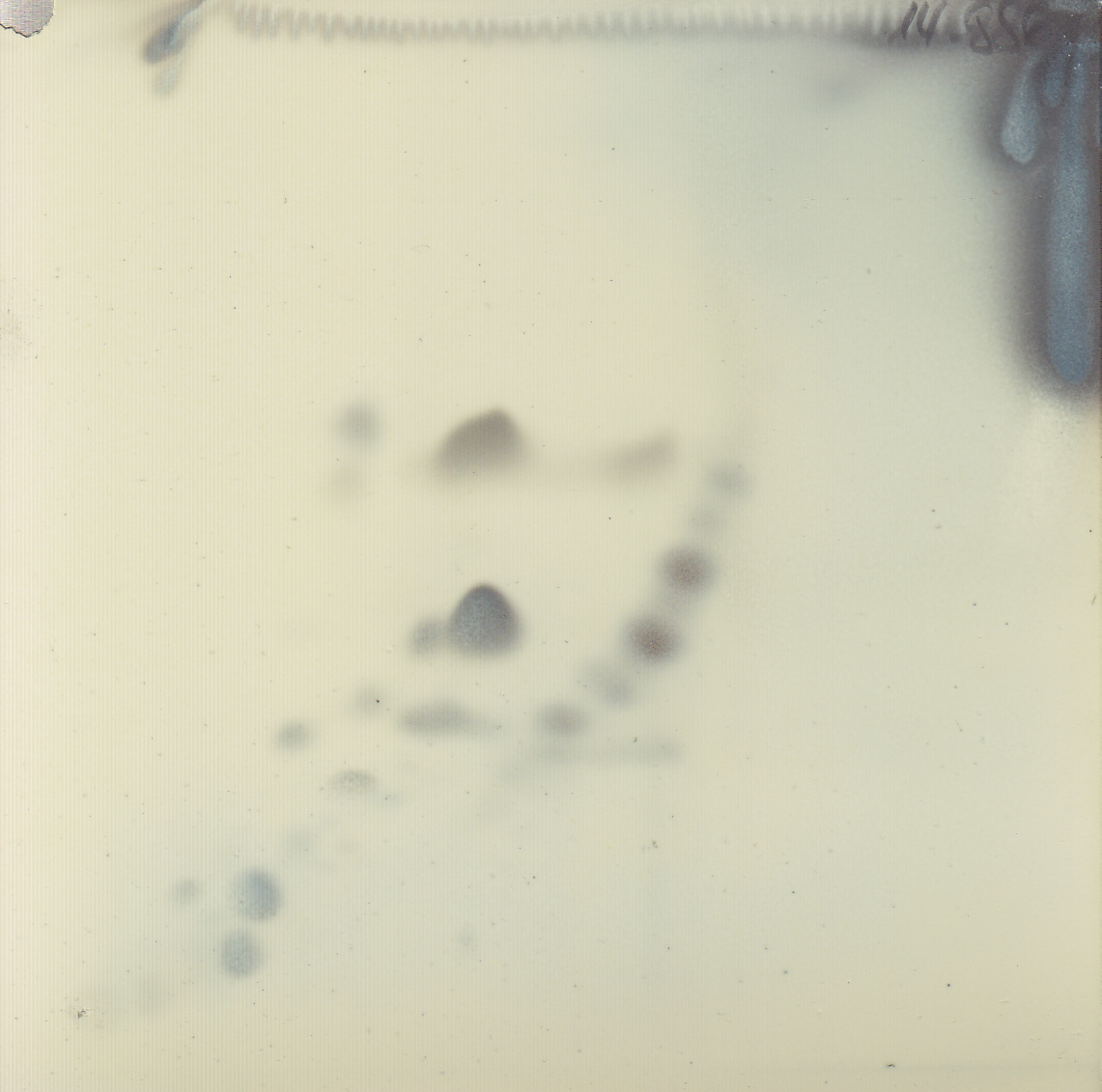
**


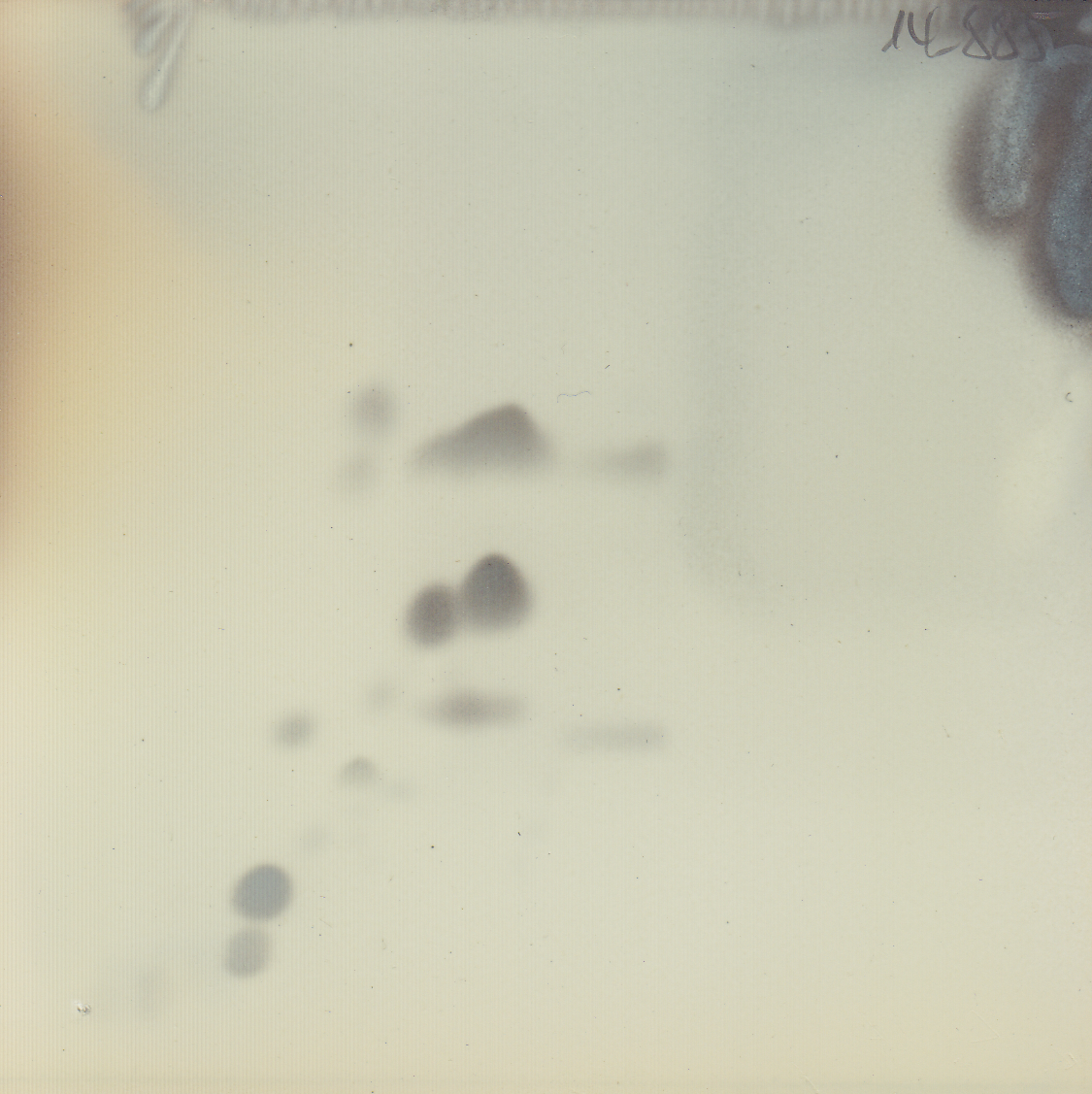


**Strain MUSC 136^T^**

**(a)**

**DPG**

**PGL**

**GL**

**PE**

**PGL**

**L**

**PL**

**AL**

**PL**

**L**

**L**

**PL**

**PI**

***Streptomyces misionensis* NBRC 13063^T^**

**DPG**

**L**

**PL**

**PL**

**PE**

**AL**

**L**

**PG**

**GL**

**L**

**PL**

**PI**

**PGL**

**PGL**

**(b)**

**Fig. S4.** Chemical structures of constituents detected in MUSC 136^T^ extract.

**
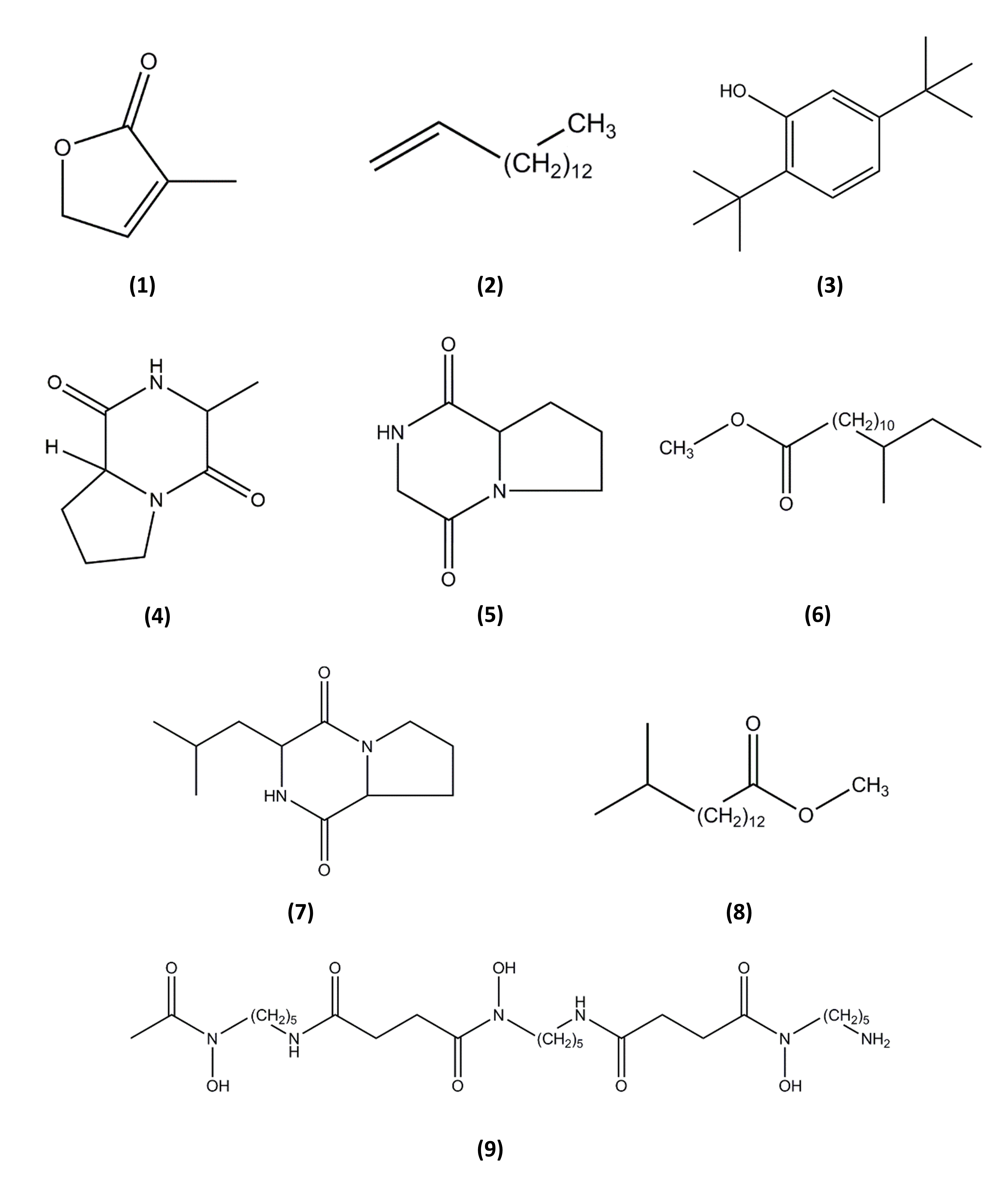
**

**Table S1.** Cultural characteristics of strain MUSC 136^T^ on different media at 28 ^0^C after 7-14 days of incubation.

-, Not detected

| **Medium** | **Growth** | **Colony color** | |
| --- | --- | --- | --- |
|  |  | **Aerial mycelium** | **Substrate mycelium** |
| Yeast malt agar (ISP 2) | Good | Yellowish white | Grayish yellow |
| Oat Meal agar (ISP 3) | Moderate | Yellowish white | Dark Grayish yellow |
| Inorganic Salt Starch agar (ISP 4) | No growth | - | - |
| Glycerol Asparagine Agar Base (ISP 5) | Good | Pale yellow | Yellowish white |
| Peptone Yeast Extract Iron agar (ISP 6) | Good | Yellowish white | Pale yellow |
| Tyrosine agar base (ISP 7) | Good | Pale yellow | Grayish yellow |
| *Streptomyces* agar | Moderate | Yellowish white | Pale yellow |
| Starch casein agar | Good | Pale greenish yellow | White gray |
| Actinomycete isolation agar | Good | Pale yellowish | Greenish white |
| Nutrient agar | Good | Yellowish white | Grayish yellow |
